# On Etherization, or the Inhalation of the Vapour of Ether

**Published:** 1847-04

**Authors:** 


### Art. XIX.

1. *A Treatise on the Inhalation of the Vapour of Ether, for the Prevention of Pain in Surgical Operations, &c.* By J. Robinson, Surgeon-Dentist, &c.—*London,* 1847. 8vo, pp. 63.

2. *Notes on the Inhalation of Sulphuric Ether in the Practice of Midwifery.* By J. Y. Simpson, m.d. f.r.s.e., Professor of Midwifery in the University of Edinburgh.—*Edinburgh,* 1847. 8vo, pp. 11.

One of the most remarkable events in the history of medicine, regarded as a practical art, is certainly that which has excited so much attention in Europe and America during the last four months,—the employment of the vapour of ether as a means of abolishing pain in the practice of Surgery, Midwifery, and Medicine. And the results hitherto obtained seem to justify us in regarding the event as no less beneficial than remarkable. It is assuredly true, that by means of the new process, not a little of that dreadful suffering, heretofore inseparable from the performance of most surgical operations, has been abolished in the practice of the most eminent surgeons in Europe and America, during the three or four months just elapsed; and there seems every reason for believing that the benefits already experienced are likely to be perpetuated, perhaps greatly enhanced in amount, through all future time. It has been the ardent desire of philanthropists in all ages to save humanity from pain, in all its forms; and the means of dissociating the practice of Surgery from one of its most terrible forms, has often formed the subject of the cogitations and the stuff of the day-dreams of the benevolent surgeon:

> ———" what best may ease
> The present misery. . . . If there be cure or charm
> To respite, or deceive or slack the pain."

The utter futility of all the attempts to attain so desirable an end by ordinary sedatives, &c. is sufficiently shown by the fact, that for many years past no means whatever have been employed by surgeons with this view, —Nature being left to sustain, as best she might, by her own unaided powers, this worst and most intolerable of human ills,—this "perfect misery," as Milton truly calls pain.

That a means should be discovered, and discovered in our own day, calculated not merely to furnish positive relief in many of these terrible inflictions, but almost to exceed, in its practical working, the wildest dreams of the philanthropic enthusiast, is a matter so much within the domain of the marvellous, almost of the supernatural, that it is no wonder we should not be yet prepared to consider it, in all its bearings, with the cool blood of philosophy. Even in this era of steam and electricity, of macroscopes and microscopes, the discovery is one that arrests more universal attention, and excites a deeper interest, than any mere physical fact whatever,—not even excepting the magnificent achievement of Adams and Leverrier, which " yields the lyre of Heaven another string."

It is but an act of simple justice to the mesmerists to admit, that they alone, among chirurgeons of the present day, have toiled for the attainment of the abolition of pain in surgical operations. This rational and benevolent aim of theirs ought to plead trumpet-tongued in their favour, while we are condemning, as we cannot fail to condemn, their manifold and monstrous aberrations from the path of common sense and philosophy. It must even be admitted, and we ourselves have admitted it—(see our 44th Number)—that the mesmerists have, to a certain extent, been successful in their aim, inasmuch as they have, in a small proportion of cases, and after a great deal of painful manipulation, succeeded in rendering patients, to all appearance, insensible to the pain of surgical operations. The evidence formerly adduced in support of this opinion has been recently much strengthened by the official report on Dr. Esdaile's experiments in the Calcutta hospital.* These attempts, however, praiseworthy as they are, will be entirely superseded, in future, by the new process of Etherization, which seems to possess infinitely greater advantages than the mesmeric process, without any of the great disadvantages of this.

We have already said that the time is not yet arrived for the calm and full consideration of this great discovery,—the true character, and value, and bearings of which can only be fixed after much more, and more varied experience than at present exists. On the present occasion, we purpose merely, by a few hurried observations, to do something towards meeting the desire for further information which our readers no doubt possess, and for the gratification of which, in some degree, at least, they may naturally look to the pages of this Journal. On some future occasion, we hope to lay before them something more worthy of them and of ourselves.

There seems to be little or no question as to the fact, that we are entirely indebted to Dr. Charles T. Jackson, of Boston in America, for the virtual, if not the actual, discovery of the use of Ether, as a means of destroying pain in surgical operations. To him, and to his coadjutor, Dr. Morton, we certainly owe the boon, whatever be its amount, of the practical application of the process now so universally employed. It is, nevertheless, true, that many others before them had, long since, not only imagined, but even made trial of the same or similar means, with somewhat similar intentions. We will here refer to a few of these foreshadowings of the great discovery, which are immediately accessible. Some of these, it will be seen, may fairly be considered as *anticipations;* but as they bore no fruit, they can only be regarded as the unvalued apples dropping idly and uselessly from the tree of knowledge: the Newtonian glance had not yet fallen on them.

In Fontana's papers, in the Philosophical Transactions, we find many experiments on men and animals, on the inspiration of different kinds of air. He himself (Phil. Trans., 1779, vol. 69, pp. 346-7,) made several experiments on himself with hydrogen gas (inflammable air). Taking it diluted, he found the process rather pleasant; but when he inspired the undiluted gas, the results were far otherwise: he became pale, confused, and fell on the floor insensible. Davy experienced precisely similar effects, but in a more intense degree, from breathing hydrocarbonate. "After the second inspiration (he says) I lost all power of perceiving external things, and had no distinct sensation except a terrible oppression on the chest. During the third inspiration, this feeling disappeared. I

* Bengal Hurkaru, Nov. 19, 1846.

seemed sinking into annihilation." (Researches, 1800, p. 468.) He was a considerable time in recovering from the effects of this dose.

Dr. Richard Pearson, of Birmingham, however, seems to have been the first, as far as we are aware, to have employed the inhalation of ether medicinally. (See his communication in the first vol. of Duncan's Annals of Med., 1796; also in vol. vii, of Simmons's Med. Facts and Observations.) He employed the remedy in phthisis and other pulmonary diseases, either simply or combined with hemlock. His mode of administering it was by inhaling it as it evaporated in an open vessel, simply by the mouth, or through an inverted funnel; or by holding a handkerchief wetted with it near the mouth and nose.

Dr. Beddoes also, in his work on Factitious Airs, published at Bristol in 1795-6, in five parts, among many other communications on the subject of inhalation of various kinds of airs in diseases, gives several communications from Dr. Pearson, on the inhalation of ether. In Part III of the same work, p. 40, there is a letter from one of Dr. Thornton's patients, in which the patient himself gives an account of the inhalation of ether, by Dr. Thornton's advice, and its effects in a case of pectoral catarrh. He says it gave almost immediate relief both to the oppression and pain in the chest. On a second trial, he says he inhaled two teaspoonfuls of ether, which, he adds, "gave immediate relief as before, and *I very soon after fell asleep*, and had a good night's rest." In Part IV, another curious case is given by Dr. Thornton, in which inhalation was prescribed for the relief of a very painful inflammatory affection of the mamma, and with very beneficial effect. In this case, however, the ether was used rather as a means of depriving the air of its oxygen, than as a direct agent; the effect of the inhalation on the patient being, as in the experiments of Fontana and Davy, to produce partial asphyxia. The results, however, are curious, and not without interesting relation to the present subject of discussion. Dr. Thornton says:

> "I therefore filled a bell-glass with atmospheric air, and burning two tablespoonfuls of ether in it, I rendered it chiefly azote and inflammable air. She persisted in inhaling this for about five minutes, standing up, until the pulse was obliterated; the eyes became dim and no longer represented the objects of vision; the face was deadly pale, and swooning coming on she fell into the arms of a servant. . . . . In about ten minutes she revived. The pulse was feeble and only 98; and for the first time, she said, for some weeks, she felt her breasts cold and easy." (p. 154.)

At this time, and subsequently, Dr. Thornton was in the common habit of administering the vapour of ether to his patients, among other pneumatic means used by him; and we are informed by one of the most eminent philosophers of our time, that he himself had this remedy administered to him in his youth by Dr. Thornton. In Mr. Robinson's pamphlet, p. 15, Dr. Boot refers to a case in which it was used in the beginning of the present century, by the late Dr. Woolcombe of Plymouth. No doubt, its use was not very uncommon at the end of the last century, though, after a time, like most other fashionable medicines, it fell into disuse and was forgotten.

In all these trials, no one had directly in view the removal or abolition of pain, though this was attained indirectly in Dr. Thornton's case. But Sir Humphrey Davy, who, it is well known, first began his chemical career by assisting Dr. Beddoes in his pneumo-medicinal researches at Bristol, seems not only to have contemplated such a result by means of medicamentous inhalation, but to have actually put it to the test of experiment on himself. The medium of his experiment, however, was not ether, but the nitrous oxyde. Sir Humphrey tells us that on two occasions the inhalation of the nitrous oxyde removed headache. He also tried its effect "in removing intense physical pain" while he was cutting a wisdom-tooth. He says, "the pain always diminished after the first four or five inspirations; the thrilling came on as usual, and uneasiness was for a few minutes swallowed up in pleasure." (Researches, p. 465.) In a subsequent part of the volume Sir Humphrey adds this remark, rendered very striking by recent events:

" *As nitrous oxyde, in its extensive operation, appears capable of destroying physical pain, it may probably be used with advantage during surgical operations* in which no great effusion of blood takes place." (Researches on Nitrous Oxide, p. 556.*)

In the article *Ether* in the 'Dict. des Sc. Med.,' vol. xiii, published in 1815, we find the author, Nysten, speaking of the inhalation of ether as familiarly known, and as employed for the relief of some pulmonary diseases, and also *for mitigating the pain of colic*, &c. He even describes (p. 385), an apparatus for the purpose of inhaling it, which closely resembles some of those now employed. It is also well known, that many years ago it was not uncommon in some of the pharmaceutical lecture-rooms and chemists' shops in London, for the pupils to inhale ether-vapour as a sort of substitute for the nitrous oxyde, or laughing gas.

To persons acquainted with these various experiments and observations, and more especially with those of Pearson, Thornton, Davy, and Nysten, the advance to the perfect discovery of ethereal inhalation, as a means of destroying pain in surgical operations, would seem but a very small step; yet this step was not made till the present day; and there seems every reason for believing that it was first made, as we have already stated, by Dr. Charles Jackson. We extract the following account of Dr. Jackson's earliest proceedings in this matter, from the 'Boston Daily Advertiser' of March 1st, 1847. After some preliminary remarks, among which we find the important acknowledgment that he was "early impressed with the remarks of Davy, concerning the remedial agency of gaseous matters," he proceeds as follows:

" In my first successful experiment the conditions as above stated [viz. that the ether should be perfectly pure and the vapour mixed with a due proportion of air] were fulfilled, though the mode of administration was of the simplest kind, it is true, but yet efficient. A folded cloth saturated with the highly rectified ether was placed over the mouth, the air being drawn freely through it, and the inhalation was continued until I lost all power over myself and sank back in my chair in a state of peculiar sleep or reverie. I experienced at first a sense of coolness, then of exhilaration and warmth followed by loss of consciousness. But it was not until a subsequent trial that I became aware that this loss of consciousness was accompanied by insensibility to pain; and a severe bronchial irritation produced by the inspiration of a large quantity of chlorine gas was for the moment relieved, and the peculiar distress occasioned by that gas was not felt, so long as

* This first and very striking work of Davy contains an immense number of experiments on himself in regard to the inhalation of different kinds of air. It well merits the attention of the profession at the present time.

I was under the influence of ether, though as that passed off it returned. I had several times occasion to mention these facts to my friends, and it is now a year since I urgently advised Mr. J. Peabody, who was associated with me as a pupil in chemistry, to inhale the ether vapour as a means of preventing pain, which would arise from the extraction of two of his teeth. He consented to try the experiment, and was preparing some ether for the purpose, but on consulting the works in which the effects of ether are mentioned, he found all the authorities arrayed in opposition to my views, and that they warned against its inhalation, as I have before stated, and he therefore did not complete the experiment.

"About the last of September or early in October last, I communicated my discovery to Dr. W. T. G. Morton, an enterprising and skilful dentist of this city, whom I occasionally advised, and who called at my laboratory to borrow an India rubber bag, which he said he intended to fill with atmospheric air, and to cause a refractory patient to breathe it, hoping to act on her imagination, and induce her to allow him to extract a tooth. I dissuaded him from this attempt, and explained to him that I had discovered a process by which real insensibility to pain might be produced. I showed him sulphuric ether, and described the method of administering it, and also its effects on the system, assuring him, that if my directions were carefully followed no danger would ensue. I advised him to try its effects on himself, in order that he might better understand its mode of operation. He followed my instructions and was successful in the first trials, in the extraction of teeth unattended with pain, the results proving exactly as I had predicted. I also furnished him with a large glass flask with a bent glass tube as an extempore inhaling apparatus. I then proposed to him the trial of the ether in a surgical operation at the Massachusetts general hospital, where it was administered by Dr. Morton, and it proved successful; but some persons who witnessed the first operation doubted the entire freedom from pain, since the patient said 'he felt a scraping.' I was therefore desirous of testing it in a capital operation, the severity of the shock being the best test with regard to the degree of insensibility. Dr. J. C. Warren politely consented to have the trial made, and its results proved entirely satisfactory, an amputation having been performed under the influence of etherial vapour without giving any pain to the patient."

Since this time our readers need not be told how widely the practice has spread, and in what a countless number of cases it has been applied in every country, and applied, we may add, with an uniformity of success and safety, which, considering the nature of the process, is most extraordinary.

The modes hitherto adopted for applying the ether vapour are very various, and the forms of apparatus innumerable. The essential points to be regarded seem to be—1, that the ether be very pure; 2, that the tube conveying the vapour be sufficiently wide to admit a current large enough to fill the respiratory organs without effort; 3, that the vapour be mixed with a sufficient proportion of air to render it easily respirable, yet not so much diluted as to render it long in producing insensibility; 4, that the apparatus possess a means of regulating this proportion accurately, the vapour being always given comparatively weak at the commencement, so that the glottis and lungs be not over-irritated; 5, that the full strength of the diluted vapour be applied as speedily as it can be tolerated by the air-passages, the nostrils being then closed, so as to exclude all extraneous air; —a strong dose, rapidly given, seems to be at once the safest and most successful proceeding; 6, that inspiration be continued, within certain limits, until complete insensibility is attained, as evinced by obvious signs; and, when stopped, to be renewed, for short periods, as often as signs of awaking, manifest themselves during the operation.

The period of time required to produce the full effect of perfect sleep and insensibility, varies considerably in different individuals; but much more, we believe, from difference in the mode of administering the agent, than from difference of individual susceptibility. When the apparatus is good, the ether pure, and the process directed by an experienced manipulator, the average period of inhalation to produce insensibility, may be stated at from two to four minutes: in a few rare cases double or triple this amount of time is required. As might be expected, children are sooner affected than adults; and, generally speaking, the influence is more perfect and more benign with them. It has frequently appeared as if the period of inhalation was much longer than what is just stated; and also that a considerable number of persons are altogether unsusceptible of the soporific influence. We are led, however, from a good deal of observation and investigation, to believe that nearly all these supposed exceptions are owing to imperfect administration of the agent. And we are inclined to think, that there are but very few persons indeed unsusceptible of this peculiar effect of ether. A general impression exists among operators, that persons much addicted to strong drinks, especially spirit-drinkers, are altogether refractory to the agent, or require a much larger dose to become affected. This may probably be true; but we have only met with two or three cases, during our investigations, which seemed to authorize this belief. In one case in which the ether was administered by a skilful and experienced manipulator, nearly half an hour elapsed before the individual came fully under the influence of the ether; this gentleman was accustomed to drink a bottle of wine or more per diem. In another case, that of a man who confessed that he took daily half a dozen glasses of gin or more, the soporific effect was not at all experienced after an inhalation of twenty minutes; although, in this case, the absorption of the ether into the blood was evinced by the *exhalation* of the etherial odours for a day or two after the trial. It is to be remarked, however, that it is not solely *the amount* of ether inhaled that regulates the supervention of the soporose state, but the amount absorbed within a given time. It is quite possible to inspire three, four, nay ten times the quantity of ether capable of producing sleep, without this state being induced, provided the vapour be taken in an extremely diluted form; and we believe that this over-dilution of the vapour and its consequent protracted inhalation, is a frequent cause of the *excitement* which supervenes so often in the practice of some persons, while it shows itself so very rarely in that of others. In these cases the patient may be made *drunk*—drunk in the first degree, but not *dead-drunk,* the condition required for chirurgical purposes.

The occasional occurrence of this *excitement* instead of the desiderated stupor, is regarded by some surgeons as a fatal objection to the practice. But we think this opinion is erroneous. In the first place, we believe that the excitement occurs extremely rarely when the process is properly regulated—probably not more than once in a hundred instances; and, secondly, there is no necessity for the surgeon operating at all in the cases in which it does occur in spite of all precautions: he may still have recourse to the old practice of operating on his patient awake. Our own observation, however, and the opinion of those who have had by far the greatest experience in the practice of etherization, lead us to believe that the proportion of persons in whom a state of excitement will frustrate operations, is extremely small.

The average duration of the state of sleep or insensibility, may be stated to be about the same as the period required to induce it—or a little less, say from two to four minutes: the period, however, occasionally greatly exceeds this, extending sometimes to half an hour, or even an hour. The awaking is generally sudden and complete; and, in the great majority of cases, the only effects it leaves behind are—a slight feeling of *muzziness* in the head, sometimes amounting to headache, and the odour and taste of ether in the mouth and nasal passages.

The immediate and obvious effects of etherization on the individual hardly require notice, as they must be familiar to all our readers, if not from personal trial at least from observation on others. All the usual phenomena of the deepest sleep supervene almost suddenly, gliding often into the profoundness of sopor, and verging occasionally upon, if not actually lapsing into, coma. The voluntary muscles become suddenly relaxed, the jaw falls, the arms hang down, the eyes roll upwards under the upper lid, the respiration becomes slow and laboured, and the face often becomes either pale or morbidly flushed. The aspect of things is, indeed, such as can hardly be contemplated, for the first time, without alarm: the individual seems, to the common eye, to be sinking into the sleep of death.

The actual effects of etherization on the functions, fluids, and organs of the body have not as yet been thoroughly investigated. Medical men have been hitherto so absorbed in the contemplation of the practical results, that they have had but little leisure or inclination to inquire into the philosophy of the thing. It may be stated, however, that the pulse is at first accelerated, and afterwards falls, but rarely to the natural standard; the respiration seems commonly to follow the same rule. The iris seems to be generally expanded, sometimes contracted.

In the state of perfect etherization we believe all sensation is abolished; in a less perfect state, an obscure perception of external objects remains, while the sense of pain is extinct. The psychical state is various. Generally speaking the sense of external impressions becomes at first confused, then dull, then false, with optical spectra or auditory illusions, general mental confusion, and then a state of dreaming or utter oblivion. In the majority of cases, the mind is busy in dreaming, the dreams being generally of an active kind, often agreeable, sometimes the reverse, occasionally most singular; and, frequently, a great deal is transacted in the few short moments of this singular trance. Many of the patients who have undergone the most dreadful operations, such as amputation of one or both thighs or arms, extraction of the stone, excision of bones, extirpation of the mamma, have readily detailed to us, and most with wondering thankfulness, the dreams with which, and with which alone, they were occupied during the operations. The character of the dreams seemed to be influenced, as in ordinary cases, by various causes, immediate or remote, present or past, relating to events or flowing from temperament:

> "Et quoi quisque fere studio devinctus adhæret,
> Aut quibus in rebus multum sumus ante moratei,
> Atque in ea ratione fuit contenta magis mens;
> In somnis eadem plerumque videmur obire."

A good many seemed to fancy themselves on the railway amid its whirl and noise and smoke; some young men were hunting, others riding on coaches; the boys were happy at their sports, in the open fields, or the filthy lane; the worn Londoner was in his old haunts carousing with his fellows; and our merry friend, Paddy, of the London Hospital, was again at his fair, wielding his shelala in defence of his friends. Others, of milder mood, and especially some of the women patients from the country, felt themselves suddenly transported from the great city and the crowded hospital-ward to their old quiet home in the distant village, happy once more with their mothers and brothers and sisters. As with the dying gladiator of the poet, the thoughts of these poor people—

"Were with the heart, and that was far away."

Some seemed transported to a less definite but still happy region, which they vaguely indicated by saying they were in heaven; while others had still odder and warmer visions, which need not be particularized.*

For the purpose of obtaining information on all the points of this most interesting subject, we personally questioned all the patients in the London hospitals, who, at the period of our visits, still remained in the wards after the ether-operations. They were in all fifty-four, and the great majority had been the subjects of capital operations. They were unanimous in their expressions of delight and gratitude at having been relieved from their diseases without suffering. In listening to their reports, it was not always easy to remain unmoved under the influence of the conceptions thereby communicated, of the astonishing contrast between the actual physical condition of the mangled body in its apparent tortures on the operating table of a crowded theatre, and the really happy mental state of the patient at the time. The old story of the magician in the Arabian Tales seemed more than realized before us, the ether being like the tub of water, one moment's dip of the head into which produced a life-long vision in the dreamer's mind. We ourselves, on trying the ether, as in duty bound to do, were not favoured by any visions good or bad; our mental condition, was, if we may so speak, that of annihilation or utter oblivion; a piece seemed snipped out of the thread of vital consciousness, as if our identity had been cut in twain.

The physical and physiological changes induced in the system by etherization are, as yet, very imperfectly observed; and the rationale of its action is far from being well understood. That the ether is immediately absorbed into the blood, and thus acts on the brain and nervous centres, either directly or indirectly, is obvious enough. The absorption is well shown by the long-continued *exhalation* of the ether by the breath of the individual who has taken it. This exhalation continues a longer or shorter period, according to circumstances, from a few hours to a few days. The presence of the ether in the body is also shown by the fact that an amputated limb has been found to exhale the etherial odour long after the operation, and the same odour has been detected on dissection, in the interior of the bodies of some who have died subsequently to an operation. It is also the prevalent opinion of surgeons that the

* We made an attempt to ascertain whether the amount of dreaming, or the character of the dreams, might be influenced by the quality of the ether. We had at first some ground for believing that the dreams were more frequent and of wilder activity, when the ether was impure; but the general results failed to bear out the first impression.

arterial blood is blackened by the ether; and it would appear that in two persons who died after etherization, the blood was found fluid after death. That this last state of blood was produced by the ether, however, is extremely doubtful, as it is well known that a like fluidity of the blood often exists in cases in which no ether has been administered.* The blackening of the blood by the inhalation of ether, has been proved by M. Amussat in experiments on animals, who further observed that the natural colour of the fluid was speedily restored on permitting the animals to breathe pure air. Admitting this, it will remain a question, whether the absorbed ether acts directly on the blood, or whether the blackness arises from the partial asphyxia induced by the partial exclusion of oxygen. The same doubt presents itself in regard to the production of the sleep and insensibility. Are these effects the consequence of the direct action of the ether on the nervous pulp, or effects resulting, secondarily, from the direct action of the blood altered by the ether? Or are they the mixed result of the direct etherial action and of the action of the hyper-carbonated blood on the brain? In a word, are the etherized patients simply *dead-drunk*, or are they *partly drunk* and *partly asphyxiated?*

We have already noticed the effect of etherization on the respiration and the pulse. When complete it suspends all voluntary motion as well as sensation, and produces a relaxed state of all the voluntary muscles. Its effect in influencing the reflex, and what may be called the external automatic actions, is not yet well ascertained; but it seems to have exerted no influence in relaxing sphincters, &c. Some surgeons seem positive that the muscles divided in the amputation of limbs do not retract as under ordinary circumstances; but this, as well as the blackening of the arterial blood, is doubted or denied by other operators. That undivided muscles are generally relaxed however, has been proved in a very beneficial way by the easier reduction of luxations, and by the readier retention of the bowel within the abdominal cavity after the operation for hernia.† Admitting this were occasionally an evil, it would have still some contravening good—as the relaxation of muscles must tend greatly to facilitate the reduction of dislocations—as well as the reposition of the bowel in hernia. An instance of this last effect is recorded in the 'Gaz. des Hôp.' of Feb. 23. A case of strangulated hernia was brought to the hospital for operation: on administering the ether, the gut was returned by the simple taxis, the resistance of the muscles having been removed.

The grand effect of etherization in abolishing the sensation of pain

* Since this page was printed, we have obtained the opinion of the most eminent pathological anatomist in London, in relation to this point; he writes as follows;—"I have no hesitation in saying that fluidity of the blood in the corpse occurs so often in connexion with various morbid states that it could never be safely ascribed to the influence of ether. Among 106 post-mortem examinations which I once tabulated in relation to the characters of the blood, it was completely fluid in four, and these were; two cases of cirrhosis of the liver; a case of tumour in the cerebellum with amenorrhœa; and a case of poisoning by opium. But I have besides clear recollections of fluidity of the blood in other cases of cirrhosis,—in one or two of tetanus,—in delirium tremens,—in many instances of fever, in the general fatty degeneration of old people. It has always been in my mind that this state of the blood coincides with so great a variety of morbid changes, that I should never be able to rely on it as characteristic of anything especially."

† This was strikingly shown in a case at the Middlesex Hospital, where the bowels, after operation (without ether), owing to extreme irritability of the parts, were repeatedly protruded to a great extent. On placing the patient under the influence of ether, the protruded bowel, on being returned, remained in the abdomen perfectly quiescent.

need not be further insisted upon: this may, indeed, be said to be the sole effect for which it is employed, as all the other effects are merely contingent and subordinate. It has, however, been made a question, whether the subjects of operations are really, in all cases, unconscious of pain, or whether they are not merely *forgetful* of what happened in their etherized state, just as a drunken man forgets on the morrow the blows and bruises he had received the day before, and which were painful enough at the time. This question can only fairly apply to those states of imperfect etherization already referred to, in which the patients exhibit more or fewer of the ordinary signs of pain, such as crying, flinching, &c. Although in such cases the individuals generally deny having suffered pain during the operation, it seems not improbable that they did suffer to a certain extent, and forgot it. What renders this probable is the fact, that they have sometimes entirely forgotten, afterwards, a rational conversation in which they had engaged immediately on awaking from their trance. In the more numerous instances of entire sopor, there are no grounds for admitting the existence of suffering at all, even if we had not positive evidence in the case of the dreamer, that a very different state of feeling existed at the time.

We now come to the most important part of our inquiry,—the practical part—the true relations of etherization to medicine and surgery—the position which it ought to hold in medical and surgical therapeutics,—and, as a corollary of this, the real value of the discovery.

So long as pain is an evil and ease a good—so long, in other words, as man is man, must any means be prized that is capable of achieving the latter by the abolition of the former. As, then, the pain of surgical operations is certainly among the most terrible of its class, and as it is no longer doubtful that etherization has the power of abolishing this, what remains for our consideration is not so much,—whether this new means shall be hailed by us as a matchless and priceless discovery, and cherished and adopted as a blessed thing: this appreciation has already been made; this adoption has been consecrated by universal practice: what remains for consideration is—Whether the good is a pure good, or is counterbalanced by attendant evils of such a magnitude as to authorize us to reject it, partially or entirely? We have already said that the time is not yet arrived for giving a positive and final judgment on the merits of the case. Etherization may be said to be still on its trial, and the verdict not yet returned. We are much mistaken, however, if, from the evidence already obtained, we may not, with considerable certainty, infer what the verdict will be. The obvious, open, palpable, glorious good of etherization, is, to deliver the wretched victims of surgical disease from the additional torture of pain, while seeking the goal of health through the portals of chirurgery. The evils that have been said to accompany or follow this good have, however, been regarded by some eminent surgeons of so serious a character, as to cause them not only to reject etherization in their operations, but to denounce it publicly as a means that will be scouted from the field of practice in less than a twelvemonth! We confess that we have been surprised to hear this opinion; as we have not been able to discover in any quarter (and we have sought it in all) any rational grounds to authorize or justify it. Of the hundreds and thousands—we might almost say of the hundreds of thousands—who have taken ether to insensibility, either out of curiosity and for experiment, or for the mitigation or abolition of chirurgical pain, in America and in Europe, we have been unable to discover, after the most extended inquiries, *a single case* in which the process *certainly* produced death, or left behind it consequences of serious importance that were *certainly* attributable to it. In a small proportion of cases there have, no doubt, been some unpleasant results, such as temporary depression of the vital powers, headache, more or less considerable for some hours, and even for a day or two; hysterical excitement in women for a similar length of time; slight bronchial irritation; nausea and sickness; and some other slight affections; but the actual proportion of patients suffering even in this slight manner has been extremely small; indeed, *wonderfully* small, when we consider the indiscriminate manner in which the practice has been had recourse to, with bad ether, bad apparatus, bad manipulators, and, speaking generally, with the whole subject in the chaotic state of a new creation, the principles not understood, the practice merely tentative and experimental. That so very few and such trifling ill effects have occurred, in such a state of things, is, to us, a most convincing proof of the general safety of the practice. So far from results of this uniformly innocent complexion being those which might have been anticipated from the rash and almost universal employment of a means avowedly capable of producing others of a very different kind, it is really surprising that actual death, not once or twice, but scores of times, has not been the consequence of this etherial epidemic. We have ourselves been constantly looking for such consequences, and we are still prepared to find them; but when they arrive, if they ever do arrive, we shall still have to consider well, before condemning the ether, whether the fatal event was a necessary consequence of its use or merely an accidental result from its abuse.

It is, however, maintained by some that these fatal results have already arrived; and, at the very moment in which we write, a coroner's jury (not the best judges, by the way, of a physiological or pathological event) have decided that in one case, at least, death has been the consequence of etherization. We must, therefore, bestow some of our attention on this part of the subject.

It is well known that deaths have followed operations in which etherization was employed; and out of the number that have taken place in England, *four*, at least, have been more or less publicly attributed to the effects of ether. The four cases to which we refer are: a case of lithotomy in one of the London hospitals; a case of amputation in private practice by an eminent London surgeon; a case of lithotomy at Colchester; and a case of the removal of a tumour at Grantham. The subjects of the foregoing cases survived the respective operations about the following periods:—the first, twelve days; the second, three and a half days; the third, more than two days; the fourth, more than one day. Authentic accounts have been published of the last two of these cases, one, by the operating surgeon in the Medical Gazette of March 5th; and one in the Times of the 19th of March, as given before the coroner; while of the first two, we have taken pains to obtain all necessary details from the best authority. We will here give a brief outline of the principal events of each case.

1. In the Colchester case of lithotomy, we are told by the operator, Mr. Nunn, that the patient "recovered from its (the ether's) effects after a short time, and continued in a quiet passive state, but *without decided reaction,* for twenty-four hours. At this period he had a chill which lasted for nearly twenty minutes." Not long after this chill, there supervened a state of collapse from which the patient never rallied, though he lived after it upwards of twenty-four hours. Nothing particular was found in the body on dissection, unless we consider with Mr. Nunn, "the fluid state of the blood" and the "flaccid state of the heart" as such.

2. In the Grantham case, (removal of a large malignant tumour from the back of the thigh,) the ether does not seem to have produced the usual full effect of insensibility, as the patient not only moved at every incision, but "struggled and nipped witness's hand," and declared afterwards, that she "felt pain when they cut." The operation was a severe and long one, lasting altogether, according to the testimony of a witness, "an hour all but five minutes," and according to the operator "about twenty-five minutes, including the tying of the vessels," the wound being "about six or seven inches long." The principal witness stated that the patient "had a little brandy and water before the operation was quite over, which she swallowed readily, and a little more when she was put to bed" . . . that "when put to bed she appeared conscious;" that "shortly after, she took a little gruel and said she felt better, but spoke in a very low and faint tone of voice;" that "she seemed quite conscious during the whole time from the operation till her decease." She never rallied, however, after the operation, but lived about twenty-eight hours after it. In this case, also, the blood was found fluid on dissection, and there was some congestion of the brain: both of which states were "in witness's [a surgeon] opinion caused by the exhibition of the ether:" there was no other unusual appearance.

3. In the case of amputation in private practice, the patient, a gentleman upwards of seventy, was placed under the influence of the ether by a most experienced man, Mr. Robinson, and took about the average quantity. He does not, however, seem to have been completely affected by the ether, as he gave signs of pain, and afterwards said he had felt some pain, during the operation. The immediate effects, such as they were, went off very speedily; as the patient was able to take wine before being removed from the table, and he seemed doing pretty well for a time, though never rallying satisfactorily. He, however, lived nearly four days, presenting various anomalous nervous symptoms, among others slight recurrent delirium; the stump did not put on the healthy reparative process.

4. In the case of lithotomy in the boy, all the primary effects of the ether passed off as usual, the patient living many days and dying from the effect of local inflammation, &c., the consequence of the operation. In this case there is no mention made of fluidity of the blood, in the account of the dissection with which we have been favoured by the eminent surgeon of the hospital.

Now we put it to the candid consideration of all experienced surgeons, whether there is anything in any one of these cases, specially different, or in any respect different, from what they have repeatedly seen after severe operations performed before the employment of ether? In three of the cases we have the ordinary phenomena of "shock" or "sinking," varied as it has been ever seen to vary in different cases. In the Grantham case, a most unusually long and severe operation (and in which by the way, as well as in the case of amputation, it may be fairly questioned if the system was ever fairly under the influence of ether at all) we have scarcely any attempt at rallying, and gradual sinking to death within thirty-six hours. In the Colchester case, though the patient never rallied well, we have no decided sinking, until twenty-four hours after, when a severe nervous rigor supervened, followed by prostration ending in death in twenty-four or thirty-six hours. In the case of amputation, we have nothing like immediate sinking, but that anomalous nervous state, so well described by Mr. Travers as "prostration with excitement," eventually ending in death after five days. In the lithotomy case in the boy, we have nothing but what is witnessed every year in every hospital; feeble reaction in a bad subject followed by unhealthy inflammation and death, many days after the operation. (In our future remarks we shall not refer to this case: it does not belong to the category at all, and is included merely because the death has been publicly attributed to the ether.)

Before we make any further remarks as to the particular character of these cases, and the cause of the patients' death, we must be permitted to lay before the reader, some extracts from one of our most estimable books in surgery, published more than twenty years ago. This work is Mr. Travers's Treatise 'On Constitutional Irritation,' a classical production with which we had erroneously fancied all our surgeons were, as they ought to be, familiar.

The principal object of Mr. Travers's book is to describe and illustrate a very peculiar condition of system to which he gives the general name of *constitutional irritation,* a condition which, when it exists, modifies in a very remarkable degree, the ordinary effects of morbid influences on the system. Mr. Travers, as might be expected, draws his principal illustrations from surgical practice.

"It appears, (says Mr. Travers) that various casualties, the operations which they require, and operations for chronic diseases, are occasionally productive of a series of symptoms indicating a fatal derangement, suspension, or failure of the powers by which life is maintained. . . . . The peculiar state which they exhibit, I particularize by the term prostration. This is of two kinds—the one pure and progressive, the other marked by alternations of excitement. The first, prostration without reaction, supervenes upon a degree of shock so intense as to destroy the irritability of the vital organs. The second, prostration with excitement, is the result of a less abrupt, or less intense shock, and indicates a greater degree of vital power, the excitement being a partial evidence of the unexhausted irritability of the vital organs." (pp. 106-7.)

"The following is a category of the symptoms indicating the two forms of prostration:—

"1. Prostration without reaction is marked by universal pallor and contraction of surface, shuddering, very small and rapid pulse, astoundment of the mental faculties, generally a dilated pupil, shortened respiration, dryness of the tongue and fauces; indistinctness, and at length cessation of the pulse at the wrist; stupor, oppressed and noisy respiration, coldness of the feet and hands, involuntary twitchings, relaxation of the sphincters, confirmed insensibility, stertor, and death.

"2. Prostration with excitement is marked by the signs of languor and stupor, or drowsiness, in the commencement, to which, after a variable interval, succeed nausea, rigor, præcordial anxiety, restlessness, jactitation; a rapid and bounding pulse, oppressed respiration with frequent attempts to sigh, flushed countenance, contracted pupil, dry heat of skin, parching thirst, rejection of liquids taken into the stomach, incoherence and wildness of expression, sometimes amounting to fierce delirium. This state is succeeded by exhaustion marked by somnolency, a profuse chilly and clammy sweat, a haggard and livid aspect, a small irregular or fluttering pulse, innumerably rapid: panting respiration, passive convulsions, hiccup and subsultus, the stupor and stertor of apoplexy, and death." (pp. 111-12.)

The following are only a few of the striking cases with which Mr. Travers illustrates his subject:

1. "A lady, Mrs. S—, who, concurring, as a point of duty, with the advice of her surgeons, reluctantly submitted to the removal of a small tumour in her breast, unexpectedly, and without any apparent cause, died on the morning following the operation. It was then, for the first time, ascertained, that she had prognosticated her death." (pp. 15-16.)

2. "I saw a man, who was the subject of strangulated hernia, expire suddenly on the table, during the steps preliminary to the operation, which, from the state of the symptoms, and of the bowel, as ascertained by examination after death, might be said to afford the fairest prospect of relief." (pp. 17-18.)

3. "A man of colour, of middle age, rather above the common stature, robust, and apparently in good health, was received into the London Hospital, labouring under a moderate sized aneurism of the femoral artery. An operation was proposed to him, to which he readily assented. On entering the theatre, however, he fainted; some wine and water was given to him, which he distinctly swallowed, and the operation was proceeded in, the artery exposed, and the ligature applied, but not tightened. During the operation, it was observed, that no pulsation could be felt in the tumour, but this was accounted for by the fainting. Before tightening the ligature, it was suggested by the operator to wait until the pulsation was re-established: some increased attention was then paid to rouse the dormant energies of the patient, and, it was remarked, that the syncope had continued an unusual time. After the attempts had been some time persevered in, a more attentive observation proved that he was quite dead." (p. 18.)

4. "A man who had been bitten in the finger by a cat, and in whom symptoms resembling those of hydrophobia had been present for twelve hours, being in perfect possession of his mind, summoned an extraordinary resolution to command his spasms, while the excision of the bitten part was performed, and died, evidently exhausted by the effort, in three minutes." (p. 19.)

5. "Sir Astley Cooper, in his Lectures, relates the case of a brewer's servant, a man of middle age, and robust frame, who had suffered much agony for several days, from a thecal abscess, occasioned by a splinter of wood penetrating beneath the nail of the thumb, and who, a few seconds after the matter was discharged by a deep incision, raised himself by a convulsive effort from his bed, and instantly expired." (pp. 19, 20.)

6. "A man whose hand was amputated for a diseased wrist-joint, supported the operation with firmness, and was proceeding in all respects well, when, on the third day from the operation, he suddenly expired. On examination, the lungs were found to contain numerous small tubercles in an incipient stage; and a recent copious effusion of serous fluid had taken place betwixt the tunics of the brain." (p. 24.)

7. "A young man, the subject of amputation of the glans penis and preputium for a cauliflower excrescence of those parts, died in five days, without any manifestation of acute disease. His lungs were discovered, on inspection, to be sprinkled with minute tubercles in the first stage of their formation, of which no suspicion existed." (p. 24.)

8. "A child, three years old, was the subject of lithotomy at St. Thomas's hospital, in the summer of 1805. The operation was admirably performed, and did not exceed one minute by the watch. A slight shivering came over the patient on being replaced in bed, and the natural temperature of the surface was not restored. He was inclined to doze, and a little convulsed, and at two o'clock the following morning died. Although this child suffered considerably from the disease, he was otherwise healthy, and his death, which excited much surprise, was attributed to fright." (p. 89.)

9. "In December, 1807, a child, aged three years and a half, underwent the same operation under as favorable circumstances. An hour after being put to bed, he also chilled; a stupor came over him, but without convulsion; and he gradually sunk into a state of deliquium, and died before ten o'clock the same night." (ib.)

10. "A lad of sixteen was cut at St. Thomas's. Everything went on well in the theatre; the same chilliness and torpor ensued, he sunk rapidly, and died at nine o'clock the same evening.

"In each of these cases, [8, 9, 10] the unfavorable symptoms showed themselves about an hour after the operation; all of them watered, but not so abundantly as usual.

"In neither of the preceding cases was the calculus remarkably large, nor had any unusual hemorrhage or other untoward circumstance occurred during the operation." (p. 90.)

11. "In January, 1808, a young and delicate child was cut for the stone, at Guy's hospital. The operation was favorable, but symptoms of irritation, and ultimately, a state of stupor succeeded, and the child died on the morning of the third day, in strong convulsions." (p. 91)

12. "In 1822, a fine boy of eighteen months, from Essex, was the subject of a private operation for lithotomy. The stone, which was obling, was easily extracted. A somewhat freer hemorrhage than ordinary occurred at the moment of the incision, but it was immediately restrained after the removal of the stone, and was too inconsiderable to create anxiety. The child, although somewhat languid and drowsy during the remainder of the day, wetted freely, and passed the night without complaint; but early on the following morning was attacked with convulsions, and died suddenly. In the afternoon of the same day, the body was minutely and carefully examined; the incision of the prostate was clean and smooth, the bladder healthy, and no morbid appearance whatever presented itself." (p. 92.)

Now, if any three, or any one of these cases had taken place after the administration of ether, and had occurred in the practice of a surgeon of the *post-hoc* school, we should like to know what judgment would have been passed upon them by the operator or by the scientific coroner and his erudite jury. Of course, the ether would have borne the blame; and the medical witnesses might, we are certain, have found, in more than one of the cases, corroborative proofs of their opinions in "congestion of the brain" and "fluidity of blood." But Mr. Travers, knowing, as he does, both the science and the history of his art, is content to let nature bear her own burthen. A great surgeon, moreover, can afford, if we may so speak, to see his patients die in the common way of surgery, without thinking it necessary to look for some new-fangled excuse among the innocent precursors of his knife. Surgery and medicine are full of these scape-goats of ignorance and incapacity, and we fear they are not likely to be soon banished from their borders.

To the foregoing cases from Mr. Travers's work, we could, from our own stores, add others, showing this tendency to sinking in as striking a point of view, under even slighter injuries. But who has not met with similar instances? Who has not seen or had authentic accounts of individuals dying after the removal of even a small common steatomatous tumour;* nay, after the extirpation of a corn? And, truly, when we look into the records of surgery, and see the vast proportion of individuals who die within a longer or shorter period after capital operations, we cannot refrain our special wonder, when we see even intelligent members of the profession frightened from their propriety by the two or three deaths that have followed operations under ether. The following extract from a paper published in the 'Edinb. Monthly Journ.,' for Jan. 1846, being the report of a speech of Professor Simpson, of Edinburgh, will perhaps teach some of our learned coroners and wise juries, and perchance even some surgeons, a little more caution in drawing inferences as to the fatal effects of etherization in chirurgical operations. We should like to know in what exact proportion of the astounding number of deaths here recorded, the fatal event took place within four or five days, or even one day, after the operation. We should also entertain a slight suspicion that, were the dissections of these cases recorded, a few *might* have exhibited both "congestion of the brain," and "fluidity of blood," although they took place before the epoch of etherization.

"Out of 89 cases in which ovariotomy had been either performed or attempted, 34 sunk, or nearly 4 in every 10 patients died.

"Out of 65 cases, collected by Dr. Cormack, in which the operation had been perfected, 25 died, or between 3 and 4 out of every 10 patients were lost.

"Now Malgaigne has shown, that out of 852 amputations of the extremities of all kinds (including those of the fingers and toes), which were performed in the Parisian hospitals from 1836 to 1841, 332 died, or about 4 out of every 10 proved fatal.

"Among these, out of 201 amputations of the thigh, 126 died, or 6 in every 10.
... ... 192 ... leg, 106 died, or $5\frac{1}{2}$ ... 10.
... ... 91 ... arm, 41 died, or $4\frac{1}{2}$ ... 10.
Of the amputations of the thigh, in 46 cases the operation was performed for severe injury of the limb: of these 34 died, or more than 7 out of every 10.

"When we looked to the results of amputation nearer home, the results were not much more encouraging. In the Glasgow Infirmary, from 1795 to 1840, Dr. Lawrie has shown that out of 276 amputations performed, 101 proved fatal, or nearly 4 in 10 died.

"Among these, out of 128 amputations of the thigh, 46 died, or $3\frac{1}{2}$ in every 10.
... ... 62 ... legs, 30 died, or 5 ... 10.
... ... 53 ... arm, 21 died, or $4\frac{1}{2}$ ... 10.

"In the Edinburgh Infirmary, during the four years commencing July 1839, there occurred 72 amputations of the thigh, leg, shoulder-joint, arm, and fore-arm. Of these 72 patients, 37 recovered and 35 died,—or nearly 5 in every 10. Of these amputations, 18 were primary. Out of 4 primary amputations of the leg, 1 patient recovered and 3 died. Out of 4 similar amputations at the shoulder-joint, 1 recovered and 3 died. There was one primary amputation of the arm; the patient died. There were eight primary amputations of the thigh; all the eight patients died. (See Dr. Peacock's Official Reports.)

"Mr. Phillips has collected the histories of 171 cases in which the larger arteries of the body were tied: of these 57 died; or about $3\frac{1}{3}$ in every 10. Dr. Inman

* Every one has heard of the death of a celebrated lady of rank, within a few hours after the removal of a small tumour from her head, by Sir Astley Cooper.

has collected 199 cases of these operations; 66 died, or about 3⅓ in every 10. Out of 40 cases of ligature of the subclavian artery which he has tabulated, 18 proved fatal, or nearly 5 in every 10 died.

"In his work on Hernia, Sir A. Cooper records 36 deaths among 77 operations for that disease, or nearly 5 in every 10 died. Dr. Inman has collated 545 cases of operation for hernia; 260 proved fatal, or nearly 5 in every 10 of the patients died.

"In the earlier years of life lithotomy is comparatively a safe and legitimate operation, and few die. But it is quite different when the operation is submitted to at 40 years of age, and upwards. At and above this term of life, Dr. Willis has shown, from numerous statistical returns, that from 2 to 5 out of every 10 operated upon die.

"Even what we deem slighter operations, are sometimes attended by no inconsiderable danger to life. Out of 95 cases of excision of the mamma, referred to in Dr. Cormack's Journal for February 1843,—20 died, or 2 in every 10. In how many cases of the remaining 75 would the disease inevitably return and ultimately destroy the patient?

"Ovariotomy then is fatal in the proportion of about 35 or 40 in every 100 operated upon, but in most capital operations we singly have as high or even a higher mortality than 35 or 40 per cent. Amputation of the thigh is higher. So is amputation of the arm. Ligature of the subclavian, for aneurism, is higher. Tying the innominata is fatal in every case. The operation for hernia has a higher mortality. Lithotomy is as fatal in most hands after the middle term of life. Even amputation of the leg below the knee is scarcely more safe, or at all events as many, or more, die after amputation of the leg, in the hospital practice of Paris and Glasgow, as die after ovariotomy."

Keeping in view the facts now detailed, and the inferences to which they obviously lead, we may state, in a very few words, the merits of the question at issue.

On the one hand, we have three cases out of the many hundred of etherized patients subjected to capital operations, in which the patients, after the departure of all the peculiar effects or primary symptoms produced by etherization, such as sopor, insensibility, &c., succumbed with a set of other and equally peculiar secondary symptoms, well known to surgeons, well known to terminate frequently in death, and, in the cases in question, *presenting not one peculiarity to distinguish them from the old ordinary cases of "sinking from shock."* On the other side, we have many thousands of instances in which the same process of etherization was had recourse to, for slight operations, such as extraction of teeth, or for mere experiment, and in which all the same primary phenomena of etherization were as effectually induced as in the others, and yet, *not one example of the occurrence of the peculiar secondary symptoms referred to,* much less any instance of death. If, in the three fatal cases, the etherization was, in any way, the source of these peculiar symptoms, or the cause of death, is it not most extraordinary that in the other thousands of instances, it should not have given rise to some of the symptoms which in these three cases preceded death, or even to death itself? What was the *sole peculiarity* that existed in the two sets of cases that had relation to the process of etherization? Only the important one, that in the set in which the deaths supervened, there was a severe operation, and in the other there was not. Is not the conclusion irresistible—that in these fatal cases, *it was the operation, not the ether which killed the patients?*

In adopting this conclusion, we beg to guard ourselves against the imputation of denying that the process of etherization can be productive of injurious or even fatal results. We believe that it both can and will be productive of both. In what we have stated we have been merely attempting to show, that, according to legitimate reasoning, we are not justified in attributing the particular deaths in question, to etherization. It is not, moreover, in the way in which it has been presumed to act in these cases, that we expect ether will be injurious ; but in its immediate and primary effects, as by inducing axphyxia and coma, from improper or excessive administration. As we have already said, we are surprised that events of this kind have not already presented themselves, more especially as the most impure alcoholic ether has been used by some operators, and a truly axphyxiating apparatus (viz., the common bladder and stop-cock, without a valve,) by others. The very originator of the plan, Dr. Jackson, not only contemplated such results, but even considered beforehand the best means for remedying the evils when they should occur. He recommends the inhalation of oxygen gas. But who has oxygen gas always at hand? And if we wait to prepare it, our patient may probably be dead. Sir H. Davy took nitrous oxide when he had nearly axphyxiated himself. We believe the open air, sprinkling with cold water, and, if necessary, the artificial inflation of the lungs by common air, are all that will be requisite, or that can be done. Very probably, also, individuals will be met with of such peculiar idiosyncrasies as to suffer injuriously, and even fatally, from the primary or secondary effects of the ether, even where the process was best administered. The non-occurrence hitherto of injury or death from any or all of these causes is, as we have already said, a singular proof of the innocuousness of etherization. An ingenious and learned friend of ours in Edinburgh, to whom the medical world is much indebted, and who thinks with us as to the innocuousness of ether, and the scientific authority of coroners' juries, supplies us with an amusing analogical illustration of this presumed innocuousness, which we will here give. The argument, it will be seen, proceeds on the assumption that intoxication by Scotch whisky taken into the stomach, is analogous to the intoxication produced by ether inhaled into the lungs. If the analogy is denied, the argument must fall to the ground. The fact, however, is curious, and here it is : "The other day (says our correspondent) I found on inquiry, that since Dr. Tait has been surgeon to our police here, not less than 27,000 people have been brought to the police offices *drunk*, and deeply so. Of these 27,000, *three* only have died, (except in metaphor,) and these three *from exposure to cold*, &c., *along with the whisky*. This is one death in 9,000. Now, could you give 27,000 black draughts to 27,000 patients, and show such a small list of killed and wounded? I take it that more than *three* would abscond from this life under diarrhœa. And so with regard to any other active medicine. *Intoxication*, then, would appear to be one of the safest *therapeutic* states we can induce!"

But as this is a somewhat ticklish line of argumentation for teetotallers, we must abandon it; and return to our text, with a word or two on the apocryphal deaths which have ensued from etherization, and which have been sufficiently numerous in London professional gossip. One day we had death from asphyxia; another, from coma; another from hemoptysis; some from convulsions; a few from pneumonia; and one or two from actual incremation or explosion through the accidental firing of the etherial vapour within the air-passages! We have not had time to investigate all these terrible cases; but we may state that we traced the one which seemed the best authenticated—that from *hemoptysis*—from its full-blown majesty in after-dinner gossip, to its humble source in the hospital. And this was the case, as the man himself detailed it to us: a day or to after a successful operation for hernia, under etherization, the man pricked his gums while picking his teeth with a pin: and it was the product of *this* operation, not of the ether, seen in the spitting pot by the patient's bed-side, that was bruited about town, as of itself sufficient to settle the question of etherization in all future time! *Ex uno disce.*

But although etherization has not been found actually to kill, or even to give rise to any results seriously injurious to the patient, it may nevertheless have disadvantages, of more or less importance, in relation to the result of some particular operations, or in relation to the surgeon's performance of them. We have not time to enter upon any discussion of points of this kind, and must content ourselves with merely indicating a few of them. The future experience of surgeons will, no doubt, eventually settle satisfactorily all that is doubtful in these things at present.

1. In operating upon persons completely etherized, some surgeons, from the impression which they say is conveyed to them as if they were operating on the dead body, fear that their ancient knowledge may prove at fault under these novel circumstances, and that their hands may forget their wonted cunning. But the objection is invalid. The new race of surgeons will be educated in ether, that is, if ether, as we would fain hope, is destined to become an established power—the medicamentum doloris—in the armamentarium of surgery; and, surely the erudite hand of a Liston, or of any other of our admirable operators, will soon, like the dyer's, "be subdued to that it works in."

2. Some surgeons still cling to the old notion, that the *feeling of pain*, not the mere *expression* of it, during surgical operations, is beneficial to the patient, a promotive of recovery. We will not argue with such advocates any more than with the eel-skinners. We say, and we think, with the poet, according to our own interpretation of his words,—

> "Ponamus nimios gemitus: flagrantior æquo
> Non debet dolor esse viri, nec vulnere major."

But to put out of the question all the immediate horrors, the mental misery of pain, it is surely monstrous physiology to regard it as anything less than a most enormous practical evil in surgery, and as, in itself, productive of the most grievous results. Any one who doubts this, we refer to Mr. Travers's work, already so largely quoted. He devotes one whole section to the illustration of the dangerous and fatal effects of pain. It commences with the following sentence: "Pain, when amounting to a certain degree of intensity and duration, is of itself destructive." (p. 48.) And ends (nearly) with these: "Pain, in excess, exhausts the principle of life, so that either its continuance without intermission, or the superaddition of the slightest shock subsequent to its endurance for a certain period is fatal. In operations protracted by unforeseen difficulties, as in cases of lithotomy, in which a stone is of such magnitude as to require crushing, the patient has begun to die upon the table." (p. 56.) So far, then, from deprecating the abolition of pain, on scientific grounds, in surgical operations, we are justified, by all science and all philosophy, physiological and metaphysical, as well as by experience, in regarding it as the direct source of much safety to the patients, and a fruitful preserver of life itself. And here we cannot help expressing a suspicion that the fatal results of two of the cases formerly noticed, might possibly have been obviated had the patients been placed under the full influence of ether.

Though, then, we utterly deny that pain is, in any case, useful *per se*, and maintain that it ought to be eschewed wherever it can be dispensed with, we are prepared to admit that in certain operations, it may possibly be productive of help to the surgeon, and therefore must be tolerated. The operation of lithotrity is said to be an instance in point, in which the surgeon, while extracting the foreign body, is warned by the pain, against mistaking a fold of the bladder for a fragment of the stone.

3. The process of etherization may be also inexpedient in some surgical operations, on account of the unconsciousness attending it; the voluntary acts of the patient, in obedience to the surgeon, being here conducive to the proper performance of the operation. This may be the case in certain operations about the throat and neck, and also in some other cases.

4. An objection has been made to the practice by some surgeons, on account of the influence it has on the operator's mind, by hurrying him and rendering him nervous, under the apprehension that the patient may wake up at any moment. This would be an objection of weight, if the insensibility could not be rendered sufficiently profound and permanent, which we believe it can, in almost every case.

But now, having given to surgery and to surgeons, as in duty bound, the lion's share of our article, we must, before concluding, bestow a few of our pages on medicine and midwifery.

Although certainly very promising in contemplation, and although often adopted, in various forms, by physicians, Pneumatic Medicine has never been productive of great practical benefits, and has consequently never thriven lustily with practising doctors. The most vigorous attempt ever made to establish it as a branch of practice, was that of Dr. Beddoes, at the close of the last century: but the brilliant hopes and promises of that enthusiastic genius, died with him and have not been since revived. To be sure, we have had sundry additions to his modified and factitious airs, or vapours, such as tar-smoke, iodine, chlorine, &c. &c.; and one of the immortal family of quacks is even now attempting to resuscitate in London the old favorite practice of the inhalation of "vital air," which once had so many partisans. The reintroduction of the vapours of ether into practice, under its present mighty patronage, is likely to give a new impulse to pneumatic medicine generally: we hope it will; as we are of opinion that it holds out to us not a little promise of good. Formerly, it was employed solely for its direct influence on the air-passages. It may, possibly, be still found useful in this way; but the point of view in which we are now regarding it, is in relation to its secondary effects on the nervous system and the blood, when administered so as to induce insensibility.

There are a good many cases in which this application of ether is likely to be useful; in several it has been already tried, and in some with beneficial effects. In most cases of violent pain, when the pain is persistent, it seems to deserve a trial. In pains of a spasmodic and neuralgic kind it holds out great promise of benefit; and in several of such cases, its utility has been already proved. In a case of *neuralgia* related in the 'Gazette des Hôpitaux,' the pain was removed "as if by enchantment," and did not return for some time. We have heard of similar cases in this country. In a case of *colica pictonum*, M. Bouvier, of Paris, used it with great benefit. This case had resisted the ordinary means of purgatives, warm baths, *opium*, etc., for three days. On being etherized, the patient slept forty minutes and awaked free from pain; he remained in this state for three hours, then slept for two hours more, and passed a good night. The pains did not return. (Bull. de l'Acad., 15 Feb.) *Dysmenorrhœa* is one of the diseases in which we should expect most benefit from ether. We know of four or five cases in which it has been used; and here the relief was immediate and complete. M. Bouvier tried ether in a case of *puerperal mania*, with very beneficial results. In this case there had been no sleep for a fortnight before using the ether: its use was followed on two occasions by quiet, if not sleep (un calme de quelques heures. *Ibid.*) M. Jobert also employed it in a case of simple insanity, with the effect of inducing sleep and restoring (temporarily) a state of rationality; no ill effects followed. The remedy was also tried in a case of recent hysterical mania in one of the London hospitals, with the effect of inducing sleep, after prolonged wakefulness. Do not these results justify us in expecting etherization to be an important therapeutical means in insanity?

In concluding this part of our subject, though we must still admit the general justice of the opinion given by Davy fifty years since, we trust the events just detailed justify us in believing that we have made some real progress since his time. "Pneumatic chemistry in its application to medicine is an art in its infancy, weak, almost useless, but apparently possessed of capabilities of improvement. To be rendered strong and mature, it must be nourished by facts, strengthened by exercise, and cautiously directed in the application of its powers by rational scepticism."—(Researches, p. 558-9.)

Having thus disposed of the question of etherization, as it regards the crafts of the Chirurgeon and Physician, respectively, it becomes, in the last place, our duty to endeavour to do like justice to the midwives, masculine and feminine. But, alas, if our liberality towards surgery in respect of space has made us stingy towards the doctors, we fear it must make us seem shabbier still anent our good friends the Howdies. And this is especially unfortunate and no less unfair, seeing that the very field of their daily and hourly labours is PAIN. While their brethren deal in this commodity only occasionally and as an exception, the midwives, at least in these days and these lands of civilization, live entirely on the traffic. To encourage ether, then, the queller of pain, would seem, at first sight, to ruin the trade utterly. But this is not the case here any more than in ordinary commerce; there would only be a change in the nature of the commodity, not an abolition of the remunerating process. And doubtless, our good friend, Professor Simpson, who must be held responsible for the present sacrilegious attempt to do away with the primal curse on womankind, like a legitimate and faithful son of Apollo and Lucina, as he is, was well aware of this before he set about preaching the crusade of obstetrical etherization to his brethren. And, verily, the craft is here in no danger; even if the Professor's most sanguine anticipations should be realized, which we are told go to this extent,—that fifty years hence ether will be so universal in midwifery that *pain* will be the exception not the rule, and that the mothers of future men will bring forth, not in the travail and the woe of the mortal couch, but in Elysian dreams on beds of asphodel! Be this as it may, it is certainly a matter of surpassing interest—need we not say, of delightful wonder—to know that already, by means of etherization, many women have been freed from all the pains and perils of childbed, in the hands of Professor Simpson and his followers. In a communication which we have received from Edinbnrgh, dated the 22d of March, Dr. Simpson states that he had, up to that date, used etherization some forty or fifty times, with the most perfect safety and success. We understand that he has kept it up *for hours,*—in one woman four, in another six hours,—without the fœtal heart varying above ten or twelve beats during the whole time, the mothers in both cases recovering perfectly, and both, of course, astonished at being delivered without being aware of it. We believe that Dr. Simpson, in making these statements, still inculcates caution in the use of the new means; justly regarding all his own trials hitherto, bold as they are, as merely experimental, and as only first-fruits which, however delightful and promising, may not be the positive harbingers of an abundant and a wholesome harvest.

The question of the relation of etherization to midwifery, is much more complex than in the case either of surgery or medicine. In the former, we have, generally speaking, but to consider its safety and its capacity of destroying pain; in midwifery we have to take into account numerous important muscular actions, reflex and voluntary, and to consider and determine how far these are intefered with by the ether, and whether its interference is calculated to modify injuriously, or to impede, movements essential to the parturient process. It is well known that the muscular efforts involved in the act of parturition are partly voluntary, though mainly reflex and automatic; and it must have been felt, previously to actual trial, to be a very doubtful problem, whether a means having the power to destroy, for the time, all volitional acts, might not interfere most seriously with the expulsive process. We do not pretend to be very learned in these matters; and we are not aware that the whole parturient process, has as yet been so fully and satisfactorily analysed by obstetricians as to make the present point one of easy solution. At any rate, we have not room at present to enter upon its discussion; and we will, therefore, content ourselves with remarking, as we remarked in the case of surgical operations, that, in the actual stage of the inquiry at which we are arrived, we must be more guided by experience than reasoning. Assuredly, if the parturient act could be equally well—or nearly as well—accomplished under the influence of ether as without it, and if the after results were equally good in both cases, there would be no further question as to the employment of etherization in midwifery. Our wives would have it, in spite of the doctors,—aye, whether there were law or Scripture in its favour. But the new means here, as in surgery, is only still on its trial; and it will, probably, be some time before the final verdict is returned. In the meantime, we are bound to say that all the evidence hitherto published is decidedly in favour of the safety and utility of etherization in midwifery; and we think accoucheurs are not simply justified in making trial of the ether in protracted labours accompanied with great suffering, but that it is their duty to try it—or, at least, to propose the trial of it to their patients. They may safely state the fact that, hitherto, not only no death, but no untoward event whatever, has been the consequence of the new practice, while the relief afforded by it has been uniformly great. Our present experience, to be sure, is not large, being, as far as we know, only the forty or fifty cases above referred to as occurring in Professor Simpson's practice, five cases detailed by M. Dubois to the French Academy; and a few others in our own and the foreign journals.

M. Dubois's opinion is, on the whole, not in favour of the employment of ether in midwifery, although he admits that he has seen no ill effects that he could, with certainty, attribute to it. He thinks, "that it should be restrained to a very limited number of cases, the nature of which ulterior experience will better allow us to determine." He, however, confesses that the result of the cases he has treated in this manner have lessened the fears with which be originally entered on the trial. We leave the Professor and the Baron—the doughty champions and learned representatives of the Obstetrics of Paris and Edinburgh—to fight the battle between them. Time, at least, will ere long determine which of the two is in the right. We are disposed to believe that neither is absolutely so; and that here, as in so many other instances of clashing opinions, the truth lies between.

The following are the general conclusions drawn by M. Dubois from his experience in obstetrical etherization.

"From my foregoing observations on the subject of ether, considered in its application to cases of midwifery, I feel myself justified in drawing the following conclusions:

"1st. That the inhalation of ether has the power of preventing pain during obstetric operations;

"2d. That it may also momentarily suspend the natural pains of labour;

"3d. That the state of ebriety induced by the inhalation of ether does not suspend uterine contraction when the latter is decidedly set in, and takes place at short intervals; and that it does not impede the synergetic action of the abdominal muscles.

"4th. That the state of ebriety appears to lessen the natural resistance which the perinæal muscles oppose to the expulsion of the head.

"5th. That the inhalation of ether has not appeared to exert any bad influence over the life or health of the child." (Lancet, March 6.)

We have taken no notice of the numerous and varied forms of apparatus that have been recommended and used for the administration of ether. Our readers must be sufficiently acquainted with them, as they have been almost all both described and figured in the weekly journals. The most ordinary type of these is that figured in Mr. Robinson's useful pamphlet, which has at least the merit of success in its favour, as we believe Mr. Robinson has made more extensive use of etherization in his own practice than any other dentist in London, and has also been much employed by the surgeons in administering ether in their more important operations. One on the same general plan as Mr. Robinson's, but with what we consider as an improvement in the mouth-piece, and in the regulating valves, has been introduced by Mr. Squire, and is also much used. It is that employed by Mr. Liston in University College Hospital, and also in his private practice. Mr. Startin's apparatus is on a somewhat different principle, the air having to pass through water before it becomes charged with the etherial vapour. It seems to answer well, and is the apparatus that has been chiefly employed at King's College Hospital. Mr. Tracy, of St. Bartholomew's, has introduced one on a smaller scale, which has been used successfully by the surgeons of that hospital in their numerous operations. There are many others, planned by Mr. Smee, Mr. Weiss, etc. etc. Dr. Snow's apparatus, that which has been principally used at St. George's, has the important advantage of enabling the operator to know exactly the strength of the vapour administered, and to regulate its quantity, and also to ascertain the precise amount of fluid ether consumed. For a figure of this apparatus, and for important observations on the principles on which etherization should be generally conducted, we refer the reader to the 'Medical Gazette,' of March 19.

On reaching this, which we had looked to as the goal of our labours for the present, and finding that there still remained a small portion of our allotted space unoccupied, we at first thought of filling it with a summary of the most important conclusions supplied by all that precedes, and also such general inferences and deductions as might appear to us to be justified by the consideration of the whole subject of etherization. On reflection, however, we feel that such an attempt would have the appearance of giving to what we have written an aspect of greater formality and completeness than it deserves, and tend to divest it of that sketchy and fragmentary character to which alone it can lay claim. We are, therefore, glad to be able to substitute for such generalities, which, at the present time, must be necessarily imperfect, some extracts from three important papers on our subject, which have only reached us since the preceding article was in type. The first two of these give us the views of two very eminent physiologists, on the physiological action of ether, which we have merely hinted at in the preceding pages, (see p. 555). The third is valuable, partly on the same account, but principally in a practical point of view.

### I. *Experiments on the Effect of Inhalation of Ether on the Nervous System of Animals.* By F. A. Longet.

This is the most elaborate and important memoir that has yet appeared on the subject of ether. We can only find room for a few of the general propositions in which the results of the experiments are summed up.

1. In etherized animals, there is absolute momentary suspension of sensibility, as well in all parts of the cerebro-spinal axis usually sensitive, as in the nervous trunks themselves.

6. The action of ether on the nervous system is much more directly and completely stupefying than alcohol, which merely renders the sensibility more obtuse without suspending it entirely, at least in the nervous centres.

7. Ether abolishes, momentarily, but completely, the excito-motor, or reflex action of the spinal marrow and medulla oblongata; and consequently acts in an opposite manner to strychnine and opium, which exalt it.

9. The functions of the encephalic centres always are suspended before those of the spinal marrow, and return before them.

10. Ether supplies a new means of isolating, in the living animal, the seat of general sensibility from the seat of the intellect and will.

11. In animals, we can so graduate the action of ether as to produce, at will, two stages, which I name—1, *Etherization of the cerebral lobes*;—2, *Etherization of the annular protuberance.**

13. Ether is only preventive of pain when it acts on the annular protuberance.

14. In animals which have suffered etherization of the annular protuberance, this organ always recovers its functions as the perceptive centre of tactile impressions, before it becomes itself a sensible organ.†

15. The course of the phenomena of etherization is far from being the same in men as in animals.

16. The process of *de-etherization* of the annular protuberance may begin while *etherization* of the cerebral lobes still continues: this explains the cries that take place towards the end of some operations which commence amid the most perfect quiet,—cries, however, of which the patient retains no recollection on awakening.

17. The *true chirurgical period* corresponds to that of *etherization of the annular protuberance*, or absolute insensibility.

* The following are the phenomena in animals (dogs and rabbits) which M. Longet considers as showing the etherization of these two parts respectively:

"1. In etherization, when the animal is no longer able to stand, it falls on its side stupefied, and then sinks into a profound sleep, is no longer conscious of external impressions, or able to perform any voluntary motions; though at the same time it still cries, and also winces on being pinched, but without *awaking*, so as to react, in an efficient and voluntary manner, against the external violence: this stage of the process I call *etherization of the cerebral lobes*, and of the other parts of the encephalon,* except the annular protuberance and *medulla oblongata* (le bulbe rachidien).

"2. When, in the further continuance of etherization, the animals no longer cry, or move, or feel, even when the most sensitive part of their nervous system is twitched or torn; I call this stage *etherization of the annular protuberance*, the effects of which are united to those of the preceding stage."

† Recouvre toujours son rôle de centre perceptif des impressions tactiles, avant de redevenir lui-même organe sensible.

---

* Cerebellum, tubercula quadrigemina, corpora striata, thalami optici.

18. For some time after the faculty of sensation is restored in etherized animals, there is transient exaltation of the sensibility.

20. At a particular period of the experiments, the blood becomes almost black in the arteries : *insensibility always shows itself previously to this occurrence.*

21. If, after the point of total insensibility, inhalation is continued, the animals (rabbits), *cæteris paribus*, die within the space of from six to twelve minutes.

22. On the contrary, on mixing a greater quantity of air with the vapour, the period of insensibilty may be kept up a long time (three quarters of an hour and more) without injury to the animal's life.

23. Ether introduced into the stomach does *not* produce insensibility in animals.

24. In etherization, the functions of the *ganglionic nervous system* appear to be over-excited, and this system appears to become a sort of *diverticulum* for the nervous power which has, for the time, abandoned the *cerebro-spinal system.*

25. The death of etherized animals is, perhaps, owing to a sort of asphyxia originating particularly in the respiratory nervous centre (*le centre nerveux réspiratoire.*)—*Archives de Méd., Mars,* 1847.

### II. *On the Effects of inhalation of Ether on the nervous centres.* By M. Flourens.

M. Flourens' experiments were made on dogs. After the animals were rendered insensible by the inhalation, the spinal marrow and medulla oblongata were laid bare and submitted to excitation and injury of various kinds. The experiments were—1, on the spinal marrow; 2, on the medulla oblongata; and 3, carried to the extent of producing death. The following are the conclusions drawn from each of the first two series of experiments, and also from the whole.

"Ether has the faculty of destroying temporarily, in the spinal marrow, the principle of sensitiveness* (principe du sentiment) and motion. The principle of sensitiveness always disappears first. When the effect of the ether passes off, the spinal marrow recovers its ordinary powers."

Under the action of ether, the nervous centres lose their powers in regular succession; first, the cerebral lobes lose theirs, viz., the intellect; next, the cerebellum loses its, viz., the power of regulating locomotion; thirdly, the spinal marrow loses the principle of sensitiveness and of motion; the medulla oblongata still retains its functions, and the animal continues to live: with loss of power in the medulla oblongata, life is lost.

"It is impossible [continues M. Flourens] to observe a single case of etherization without being struck with the similarity of its phenomena to those of asphyxia. I subjected two dogs to the simplest form of asphyxia, by confining them in a limited

* We purposely use this less common word, and also give the original, for fear of misleading.

quantity of atmospheric air. The result was, a state of asphyxia similar to etherization. On baring the spinal marrow, the animals felt nothing, nor did they when the sensorial portions (parties sensoriales) of the cord were pricked or cut. On pinching the motor portions, there were only a few feeble muscular contractions. There is, therefore, a real relation, a marked analogy, between etherization and asphyxia. But in ordinary asphyxia, the nervous system loses its powers under the action of black or deoxygenated blood, while, in etherization, it loses them, in the first place, under the direct action of the ether. This is the only difference; for in both, there is the same loss of sensitiveness (sentiment) and voluntary motion, the same continuance, at least for a time, of the respiratory movements, —in a word, the same survival of the medulla oblongata and medulla spinalis. In this way, etherization lays open to us the true mechanism of asphyxia, in other words, the *successive death* of the nervous centres in asphyxia. And it is in this successive progress of the death of the nervous centres, that the great value of these new experiments consists. Etherization, like mechanical experiment, isolates respectively, the intellectual faculties, the co-ordination of muscular motions, sensibility, mobility, life. In the etherized animal, one point alone—*nodus vitalis* —survives; and while it survives, all other parts live, at least, with a latent life, and are capable of resuming their complete life (*leur vie entière.*) This point dead, all dies." (*Gazette des Hôpitaux*, 20 *Mars*, 1847.)

At the sacrifice of excluding other important matter from our pages, we will also here transcribe the greater portion of a valuable paper of Dr. Snow, that has only this day (March 26) appeared in the London Medical Gazette.

We strongly recommend this paper to the perusal of our readers. Dr. Snow has paid very great attention to the whole subject of etherization, and has had much practical experience in applying the agent. The reader will observe that several passages in Dr. Snow's paper go to the elucidation of points but slightly or doubtfully touched on by ourselves in our article. One paragraph on "the psychological phenomena" will be found to have an interesting relation to the experiments of M. Longet and Flourens.

### III. *On the Inhalation of the vapour of Ether.* By John Snow, M.D.

"In those instances in which I have watched the pupil of the eye narrowly, I have observed it to dilate, as the patient is getting under the influence of the vapour. This dilatation is, however, but transitory, and the pupil usually becomes somewhat contracted, and the eye turned up, as in sleep, as soon as the patient becomes insensible to pain. The breathing at the same time becomes deep, slow, and regular, and there is an absence of voluntary motion and a relaxation of the muscles, the orbicularis muscle ceasing to contract again on the eyelids being raised by the finger. An operation may be commenced in this condition of the patient, with confidence that he will remain as passive as a dead subject. This having been found to be the case, in order to maintain the insensibility without further increasing it, I am in the habit of partly turning the two-way tap to dilute the vapour; and it has seemed to me that by turning it about half way, so as to admit an equal quantity of external air, and reduce the vapour to about 25 per cent., that object has been attained: but more extensive experience is required on this point, and perhaps the proportion required may vary in different patients. This method of continuing a more diluted vapour I have found to keep up the insensibility better than leaving off the process and resuming it by turns. But if the respiration becomes too slow, or at all stertorous, or if the pulse becomes very small or feeble, the nostrils should be at once liberated, and the admission of fresh air will afford immediate relief. I should think it unsafe to fasten a mask on the face, by means of a strap and buckle going behind the head, or to use any means that would interfere with the instantaneous admission of air, for on one occasion I saw an animal killed by ether by a momentary delay. It was placed in a small glass jar, and when it appeared to have had as much of the vapour as it could bear, I attempted to take it out, but could not reach it with my fingers, and whilst turning round for some means of extricating it, it expired.

. . . . . . .

"In nineteen cases out of twenty in which the pulse was carefully noticed, it increased in frequency during the inhalation, often very much, becoming as frequent as 180 in the minute in some patients in whom, from debility, it was frequent before the process began. Generally the pulse has also become smaller and more feeble. In one instance, that of a lady reduced in strength by malignant disease, it became smaller, but not more frequent; and as soon as the inhalation was discontinued, it became fuller and stronger than before the inhalation began. The pulse generally recovers its volume almost directly the inhalation is discontinued; in several instances, as in the above, becoming stronger than before; but it remains frequent for some minutes. . . .

. . . . .

"I have seen two cases in which the depressing effect of the inhalation was considerable, and was not followed by reaction directly it was discontinued. . . . . A lady, 41 years of age, in pretty good general health, the patient of Dr. Frederick Bird, inhaled ether on the occasion of having a tumour removed connected with the external generative organs. She inhaled for eight minutes, during which time it was observed that the respiration was feeble and slow. The pulse, however, which had been about natural before the inhalation, became feeble and very frequent, and the patient began to struggle as if suffering from want of breath; the process was discontinued, although she did not appear insensible, and the operation was commenced. She flinched and cried out at the first incision, although she did not afterwards remember the pain. She became very faint during the operation, although there was but little loss of blood, and it was necessary to give brandy, and lower the head to the horizontal posture. Consciousness soon returned, and as some sutures were made in the skin, she spoke coolly of beginning to feel a little pain. The feeling of faintness continued more or less all night, but her recovery was very good. The apparatus in this instance was placed in water at 70°, being lower than the temperature of the room. Two fluid ounces of ether were put in, and three drachms remained; consequently 13 drachms were inhaled, equal to about 709 cubic inches of vapour; and as it was washed ether, each 115 cubic inches would be combined with 100 cubic inches of air; consequently only about 616 cubic inches of air were breathed, making 1325 cubic inches of air and vapour: but in eight minutes the patient ought to have breathed about 2400 cubic inches of air alone. The ether in this instance appeared to act as a sedative to the function of respiration, and the small amount of air breathed may perhaps account for the depressing effects.

"In two or three instances there have been some struggling and a distended state of the superficial veins, the skin being rather purple, and the conjunctivæ somewhat injected. In one instance this seemed to arise from cough being excited by the vapour, on account of the bronchial membrane being in an irritable state, and in the others I believe it arose from obstructed respiration, which in future may be avoided, rather than from the direct effect of the vapour. By the kindness of the surgeons to St. George's hospital, I have had the honour of giving the vapour of ether at thirteen surgical operations—most of them important ones—in the hospital during the last six weeks, having the valuable advice of the surgeons, and occasionally also of one or two of the physicians to the hospital, to aid me in so giving it. It has been successful in altogether preventing pain in all the cases but one or two, and even in these there was but very little of the pain that there otherwise would have been; and there have been no ill effects of any kind following the inhalation of the ether. I allude to these cases to remark that five of the patients were children of various ages, from the fifth year upwards, and that they inhaled more easily than the adults generally did; that they were more quickly affected, generally becoming quite insensible in less than two minutes, and always without any of the struggling which sometimes occurred in the adults. For a variety of reasons, and from close observation, I have arrived at the conclusion, that this difference has not arisen strictly from a different effect of ether on subjects of different ages, but from a cause within our control. The same inhaler was used in all, consequently the tubes were wider in proportion for children than for adults. I have described all the passages of the apparatus as not less than five-eighths of an inch in diameter; but such is the description rather of what I wanted, than of any instrument I have used. Valves and tubes such as were already in existence have been made use of, and the caliber in some part of its extent has always been contracted to half an inch, and this I consider only enough for a child, but not for the adult. As only half, and often not so much as half, of what is inhaled is air, it is particularly requisite that the tubes should be wide. I am now getting elastic tubes, valves and mouth-tubes, made purposely for the apparatus, three quarters of an inch in diameter, as wide, in fact, as the barrel of a fowling-piece, and intend to give ether as fair a trial in adults as hitherto, I believe, it has had in children only. The pipe admitting air to the ether will be five-eighths, and all the passages for the air expanded by vapour, three quarters of an inch in diameter. It may be supposed that there is no occasion to make the tubes larger than the trachea, but something ought to be allowed for the friction of the air against the interior of the tubes.

" With respect to the psychological phenomena produced by ether, I have observed that consciousness seems to be lost before the sensibility to pain, and if an operation is commenced in this stage, the patient will flinch, and even utter cries, and give expressions of pain, but will not remember it, and will assert that he has felt none. Metaphysicians have distinguished between sensibility and perception—between mere sensation and the consciousness or knowledge of that sensation, though the two functions have, as they supposed, always been combined. Ether seems to decompose mental phenomena as galvanism decomposes chemical compounds, allowing us to analyse them, and showing that the metaphysicians were right. During the recovery of the patient, consciousness, which first departed, generally returns first, and the curious phenomenon is witnessed of a patient talking, often quite rationally, about the most indifferent matters, whilst his body is being cut or stitched by the surgeon. I have never seen this insensibility to pain during the conscious state except where consciousness had been previously suspended. In the paper on the capillary circulation, in the ' Medical Gazette,' to which I have alluded above, I offered the opinion that the pain of inflammation depended on a great increase of the natural sensibility of the inflamed part. Under the influence of ether we sometimes see the converse of this, viz., what would be pain reduced to an ordinary sensation; thus, some patients, whilst recovering their consciousness, feel the cuts of the surgeon without the smart. A nobleman, the patient of Mr. Tracy, of Hill street, Berkeley square, described the lancing of an abscess as the sensation of something cold touching the part; the manipulation of the abscess, which at another time would have been painful, he did not feel at all.

"If the patient will remain silent during his recovery from the effects of ether, as he generally will, it is better not to trouble him with questions till he has perfectly regained his faculties, as conversation seems to increase the tendency to excitement of the mind that sometimes exists for a few minutes as the patient is recovering from the effects of ether. This kind of inebriation is sometimes amusing, but is not a desirable part of the effects of ether, more especially on so grave an occasion as a serious surgical operation; and therefore anything that may prevent or diminish it is worthy of attention. The children have all appeared to recover their consciousness very quickly, and without any kind of aberration of mind.

"Any organic disease which impedes the flow of blood through the heart and lungs would seem to contraindicate the exhibition of ether by inhalation, and I should consider a hurried state of the circulation, such as that induced by strong labour-pains, likewise to offer an objection to the process.

. . . . . . .

"In concluding, however, I should wish to observe that I am inclined to look upon the new application of ether as the most valuable discovery in medical science since that of vaccination. From what I have seen, I feel justified in the conclusion that ether may be inhaled for nearly all surgical operations, with the effect of preventing pain, not only with safety and without ill consequences, where due care is taken, but in many cases with the further advantage of improving the patient's prospect of recovery; the pain of an operation forming often a considerable part of what renders it dangerous, and many patients, after ether, having seemed to recover better than might, without it, have been expected. In the amputations performed at St. George's Hospital, whilst the patients were under the influence of ether, it has been remarked, as was stated by Mr. Cutler, on February 11th, that there has been an absence of the painful spasmodic starting of the stump, which usually renders it necessary for a nurse to sit and hold it for some hours after the operation."

(Med. Gazette, March 26th, 1847.)

